# Exposure to Particulate Matter Air Pollution and Anosmia

**DOI:** 10.1001/jamanetworkopen.2021.11606

**Published:** 2021-05-27

**Authors:** Zhenyu Zhang, Nicholas R. Rowan, Jayant M. Pinto, Nyall R. London, Andrew P. Lane, Shyam Biswal, Murugappan Ramanathan

**Affiliations:** 1 Department of Otolaryngology–Head and Neck Surgery, Johns Hopkins University School of Medicine, Baltimore, Maryland; 2 Department of Global Health, The Peking University School of Public Health, Beijing, China; 3 Section of Otolaryngology–Head and Neck Surgery, Department of Surgery, The University of Chicago, Illinois; 4 Department of Environmental Health Sciences, The Johns Hopkins Bloomberg School of Public Health, Baltimore, Maryland

## Abstract

**Importance:**

Anosmia, the loss of the sense of smell, has profound implications for patient safety, well-being, and quality of life, and it is a predictor of patient frailty and mortality. Exposure to air pollution may be an olfactory insult that contributes to the development of anosmia.

**Objective:**

To investigate the association between long-term exposure to particulate matter (PM) with an aerodynamic diameter of no more than 2.5 μm (PM_2.5_) with anosmia.

**Design, Setting, and Participants:**

This case-control study examined individuals who presented from January 1, 2013, through December 31, 2016, at an academic medical center in Baltimore, Maryland. Case participants were diagnosed with anosmia by board-certified otolaryngologists. Control participants were selected using the nearest neighbor matching strategy for age, sex, race/ethnicity, and date of diagnosis. Data analysis was conducted from September 2020 to March 2021.

**Exposures:**

Ambient PM_2.5_ levels.

**Main Outcomes and Measures:**

Novel method to quantify ambient PM_2.5_ exposure levels in patients diagnosed with anosmia compared with matched control participants.

**Results:**

A total of 2690 patients were identified with a mean (SD) age of 55.3 (16.6) years. The case group included 538 patients with anosmia (20%), and the control group included 2152 matched control participants (80%). Most of the individuals in the case and control groups were women, White patients, had overweight (BMI 25 to <30), and did not smoke (women: 339 [63.0%] and 1355 [63.0%]; White patients: 318 [59.1%] and 1343 [62.4%]; had overweight: 179 [33.3%] and 653 [30.3%]; and did not smoke: 328 [61.0%] and 1248 [58.0%]). Mean (SD) exposure to PM_2.5_ was significantly higher in patients with anosmia compared with healthy control participants at 12-, 24-, 36-, 60-month time points: 10.2 (1.6) μg/m^3^ vs 9.9 (1.9) μg/m^3^; 10.5 (1.7) μg/m^3^ vs 10.2 (1.9) μg/m^3^; 10.8 (1.8) μg/m^3^ vs 10.4 (2.0) μg/m^3^; and 11.0 (1.8) μg/m^3^ vs 10.7 (2.1) μg/m^3^, respectively. There was an association between elevated PM_2.5_ exposure level and odds of anosmia in multivariate analyses that adjusted for age, sex, race/ethnicity, body mass index, alcohol or tobacco use, and medical comorbidities (12 mo: odds ratio [OR], 1.73; 95% CI, 1.28-2.33; 24 mo: OR, 1.72; 95% CI, 1.30-2.29; 36 mo: OR, 1.69; 95% CI, 1.30-2.19; and 60 mo: OR, 1.59; 95% CI, 1.22-2.08). The association between long-term exposure to PM_2.5_ and the odds of developing anosmia was nonlinear, as indicated by spline analysis. For example, for 12 months of exposure to PM_2.5_, the odds of developing anosmia at 6.0 µg/m^3^ was OR 0.79 (95% CI, 0.64-0.97); at 10.0 µg/m^3^, OR 1.42 (95% CI, 1.10-1.82); at 15.0 µg/m^3^, OR 2.03 (95% CI, 1.15-3.58).

**Conclusions and Relevance:**

In this study, long-term airborne exposure to PM_2.5_ was associated with anosmia. Ambient PM_2.5_ represents a potentially ubiquitous and modifiable risk factor for the loss of sense of smell.

## Key Points

**Question:**

What is the association of long-term exposure to the air pollutant, ambient particulate matter (PM) with an aerodynamic diameter of no more than 2.5 μm (PM_2.5_), and anosmia, ie, the inability to smell?

**Findings:**

In this case-control study that measured PM_2.5_ exposure levels among 2690 patients at intervals for 5 years, there was a dose-response association between PM_2.5_ exposure levels and anosmia that persisted despite controlling for comorbidities known to be associated with olfaction.

**Meaning:**

These findings suggest that cumulative exposure to fine PM is associated with an increased risk of anosmia.

## Teaser

This case-control study investigated the association between long-term exposure to particulate matter with an aerodynamic diameter of no more than 2.5 μm with anosmia.

## Introduction

Anosmia, the loss of the sense of smell, has a substantial effect on overall well-being, quality of life, the experience of food, and the ability to detect environmental hazards, such as fire and toxins. Patients with disruptions in their ability to smell commonly experience weight loss, decreased social interaction, depression, and generalized anxiety.^1,2,3,4^ Moreover, olfactory function is one of the strongest predictors of mortality in older adults.^5^

Despite these concerns, anosmia is an overlooked public health problem.^6^ Although estimates vary, considerable portions of the general population have anosmia. In Sweden, more than 5.8% of adults in the general population have anosmia, while 13.7% of adults have anosmia in South Korea.^7,8^ In the US, the overall reported prevalence of anosmia ranges from 10% to 23% of the entire population, accounting for tens of millions of Americans.^9,10,11^ These dramatic statistics may underestimate the prevalence of anosmia, because patients may unknowingly experience subtle changes in olfactory function, and disruptions in olfaction may occur in more than 50% of healthy adults when detailed olfactory assessments are performed.^12,13^

The causes of anosmia can be broadly subdivided into conductive (ie, physical barriers to odorants reaching the olfactory system, including allergic rhinitis or hay fever, nasal polyps, or rhinosinusitis) and sensorineural (ie, failure of the olfactory system to detect odorants, including viral infection, neurologic conditions, or head trauma).^14^ Beyond inflammatory sinonasal and neurocognitive diseases, air pollution may present an additional olfactory insult that contributes to the development of anosmia.^15^ Several studies have demonstrated an association between air pollution and olfaction.^16,17^ The unique positioning of the olfactory nerve in the nasal cavity, directly opposed to the external environmental exposures, places the olfactory system at particular risk from airborne pollutants.

One pollutant potentially associated with anosmia is ambient particulate matter (PM) with an aerodynamic diameter of no more than 2.5 μm (PM_2.5_). This class of pollutant is associated with cardiovascular diseases, cognitive decline, and overall mortality.^18^ PM_2.5_ contains a complex mixture of solids or liquid droplets containing organic compounds, metals, and dust particles that can be inhaled and directly contact the olfactory neurons that are located in the roof of the nasal cavity. Although exposure to PM_2.5_ has been associated with olfactory dysfunction, few large-scale studies have specifically examined the association of PM air pollution with anosmia across all age groups and locations.^16,17,19,20,21^ Because nonvirally mediated anosmia clinically develops over longer periods, this study focused on investigating the association between long-term PM_2.5_ air pollution and the risk of anosmia in a large outpatient-based case-control study of patients who visited the Johns Hopkins Hospital in Baltimore, Maryland.

## Methods

### Setting and Participants

This case-control study was approved by the Johns Hopkins University School of Medicine institutional review board with a waiver of informed consent. Consent was waived because, with the exception of zip codes, no patient-identifying information was collected. This study adhered to the Strengthening the Reporting of Observational Studies in Epidemiology (STROBE) reporting guideline with a completed checklist for case-control studies in epidemiology. Patients who were 18 years or older and were diagnosed with anosmia for the first time by an otolaryngologist within the Johns Hopkins Health System from January 1, 2013, to December 31, 2016, were included in this study. Selected patients did not have a diagnosis of chronic rhinosinusitis or nasal polyposis. The diagnosis was confirmed using relevant *International Classification of Diseases, Ninth Revision* and *Tenth Revision *(*ICD-9* and *ICD-10*) diagnosis codes. The time of onset of anosmia was defined as the time at diagnosis. All patients underwent facial computed tomography (CT) scan or magnetic resonance imaging (MRI) and nasal endoscopy and were without evidence of sinonasal pathology. Clinical characteristics, including demographic data, race/ethnicity, preexisting medical conditions, and socioeconomic status (SES), were extracted from the medical records of all case and control participants. We used the US Census Bureau’s American Community Survey^22^ to determine the median household income by patient’s residence zip code tabulation area (ZCTA). The median ZCTA household income was inflation-adjusted to 2016 US dollars. Control participants were selected from patients who visited the otolaryngology department based on the following criteria: alive during this time; without anosmia, chronic rhinosinusitis, or nasal polyposis; and with diseases that were not a priori related to anosmia or known risk factors for the diseases, such as smoking, alcohol consumption, diabetes, prior traumatic brain injury, or neurodegenerative disease. Four control participants per case were selected using the nearest neighbor matching strategy for age, sex, race/ethnicity, and date of anosmia diagnosis for each identified case.^23^

### Exposure Assessment

Ambient PM_2.5_ exposure levels were estimated and validated based on previously published prediction models.^24^ Briefly, we used deep-learning neural networks that incorporated meteorological measurements, land-use terms, satellite-based measurements, and simulation outputs from a chemical transport model to estimate daily concentrations of PM_2.5_ in unmonitored areas. We acquired air pollution monitoring data from the US Environmental Protection Agency (EPA) Air Quality System (AQS) (1928 monitors for PM_2.5_). Data about daily air temperature and relative humidity were retrieved from North American Regional Reanalysis with grids that were approximately 32 km × 32 km.^25^ Satellite-based aerosol optical depths were retrieved from the Moderate Resolution Imaging Spectroradiometer (MODIS), using the Multi-Angle Implementation of Atmospheric Correction algorithm method.^26^ For vegetation coverage, we used the percentage of vegetation from the National Centers for Environmental Prediction North American Regional Reanalysis data and MODIS MOD13A2, a normalized difference vegetation index data product.^27^

We fit the neural network with monitoring data from the EPA AQS. We then estimated daily PM_2.5_ concentrations from the year 2000 to 2016 for nationwide grids that were 3 km × 3 km. Cross-validation indicated that the models had a high accuracy across the entire study area. The national mean coefficients of determination (*R*^2^) for PM_2.5_ were 0.86, with a variation between 0.71 to 0.95; the mean square errors between the measurements and estimated daily values for PM_2.5_ were 1.50 μg/m^3^. We created various exposure metrics as appropriate to examine different windows of exposure, including 12-, 24-, 36- and 60-month mean PM_2.5_ concentration before the diagnosis date. For each patient, we assigned a PM_2.5_ exposure value from the nearest estimated 3 km × 3 km grid according to the zip code of the person’s residence address.

### Statistical Analysis

Descriptive statistics for patient variables were calculated using mean (SD), or frequency count (percentage), as appropriate. Conditional logistic regression models were used to determine the association between long-term PM_2.5_ exposure and risk of anosmia. We used a base model adjusted for age, sex, race/ethnicity, and state. In model 2, we further adjusted for body mass index (BMI), which was calculated as weight in kilograms divided by height in meters squared, current alcohol consumption status, and current smoking status, which may be associated with olfaction. In model 3, we added medical comorbidities (ie, medical history of hypertension, diabetes, chronic obstructive pulmonary disease [COPD], and asthma) as potential confounders of this association.

To evaluate nonlinear dose-response associations between PM_2.5_ exposure and risk of anosmia, we modeled PM_2.5_ air pollution exposure variables using restricted cubic splines with knots at the 10th, 50th, and 90th percentiles of the distribution of PM_2.5_ exposure estimates. Statistical analyses were conducted using Stata version 16.0 (StataCorp) and R version 4.1 (R Project for Statistical Computing) from September 2020 to March 2021. *P* values were 2-sided, and *P* < .05 was considered statistically significant.

## Results

A total of 2690 patients were identified with a mean (SD) age of 55.3 (16.6) years, and 1694 (63.0%) were women. Among the 538 case participants (20.0%) with anosmia, 339 (63.0%) were women, and the mean (SD) age at baseline was 54.8 (17.0) years. Among 2152 matched control participants (80.0%), 1355 (63.0%) were female patients with the mean (SD) age of 55.4 (16.5) years. Most of the individuals in the case and control groups were White patients, had overweight (BMI 25 to <30), and did not smoke (White patients: 318 [59.1%] and 1343 [62.4%]; had overweight: 179 [33.3%] and 653 [30.3%]; and did not smoke; 328 [61.0%] and 1248 [58.0%]) (Table 1). Patients with anosmia were more likely to consume alcohol at the time of enrollment, were more likely to live in an area with lower household income, and less likely to be diagnosed with hypertension or COPD compared with control participants (consume alcohol: 270 [50.2%] vs 814 [37.8%]; mean [SD] median household income: $75 927 [$32 319] vs $86 164 [$34 533]; hypertension: 162 [30.1%] vs 762 [35.4%]; COPD: 10 [1.9%] vs 80 [3.7%]). There was no difference in prevalence of diagnoses of diabetes, asthma, or environmental allergies between the 2 groups. Most patients lived in the northeastern United States (2555 of 2690 [95.0%]).

**Table 1.** Demographic and Clinical Characteristics of Participants

Characteristic	No. (%)		*P* value^a^
	Patients with anosmia (n = 538)	Control participants (n = 2152)	
Age, y	54.8 (17.0)	55.4 (16.5)	.43
Male sex	199 (37.0)	797 (37.0)	>.99
Female sex	339 (63.0)	1355 (63.0)	
Race/ethnicity			
White	318 (59.1)	1343 (62.4)	.19
African American	143 (26.6)	556 (25.8)	
Hispanic/Latino	30 (5.6)	80 (3.7)	
Other^b^	47 (8.7)	173 (8.0)	
PM_2.5_ exposure, mean (SD), μg/m^3^			
12-mo	10.2 (1.6)	9.9 (1.9)	.003
24-mo	10.5 (1.7)	10.2 (1.9)	.001
36-mo	10.8 (1.8)	10.4 (2.0)	<.001
60-mo	11.0 (1.8)	10.7 (2.1)	.002
BMI			
Underweight, <18.5	17 (3.2)	81 (3.8)	.58
Normal weight, 18.5 to <25	176 (32.7)	734 (34.1)	
Overweight, 25 to <30	179 (33.3)	653 (30.3)	
Obesity, ≥30	166 (30.9)	684 (31.8)	
Current smoking status			
Never smoked	328 (61.0)	1248 (58.0)	.11
Currently smokes	44 (8.2)	242 (11.2)	
Formerly smoked	166 (30.9)	662 (30.8)	
Current alcohol consumption	270 (50.2)	814 (37.8)	<.001
Median household income, mean (SD), US $	75 927 (32 319)	86 164 (34 533)	<.001
Comorbidity			
Hypertension	162 (30.1)	762 (35.4)	.02
Diabetes	56 (10.4)	260 (12.1)	.32
COPD	10 (1.9)	80 (3.7)	.04
Asthma	50 (9.3)	189 (8.8)	.77

Abbreviations: BMI, body mass index (calculated as weight in kilograms divided by height in meters squared); COPD, chronic obstructive pulmonary disease; PM_2.5_, particulate matter with an aerodynamic diameter of no more than 2.5 μm.

^a^ Values were calculated using χ^2^ test for categorical variables and the Mann-Whitney U test for continuous variables.

^b^ Includes Asian, American Indian, Alaska Native, Native Hawaiian, or other Pacific Islander.

Mean (SD) PM_2.5_ exposure levels were higher in patients with anosmia leading to the time of diagnosis compared with control participants at all measured estimates with a 12-, 24-, 36-, and 60-month mean concentration of 10.2 (1.6) μg/m^3^ vs 9.9 (1.9) μg/m^3^, 10.5 (1.7) μg/m^3^ vs 10.2 (1.9) μg/m^3^, 10.8 (1.8) μg/m^3^ vs 10.4 (2.0) μg/m^3^, and 11.0 (1.8) μg/m^3^ vs 10.7 (2.1) μg/m^3^, respectively (Table 1). Multivariate modeling demonstrated a direct association between PM_2.5_ exposure levels and patients with anosmia across all models. Model 1 adjusted for age, sex, and race/ethnicity; model 2 also adjusted for BMI, current alcohol consumption status, and current smoking status; and model 3 further adjusted for a medical history of hypertension, diabetes, COPD, and asthma. In fully adjusted models (model 3), the odds ratios (ORs) for the development of anosmia associated with a 5-μg/m^3^ increase in 12-, 24-, 36- and 60-month PM_2.5_ exposure were 1.73 (95% CI, 1.28-2.33), 1.72 (95% CI, 1.30-2.29), 1.69 (95% CI, 1.30-2.19), and 1.59 (95% CI, 1.22-2.08), respectively (Table 2). Increasing PM_2.5_ concentration was associated with an increased odds of anosmia in spline regression analyses, and the trend was consistent across different exposure periods (Figure). For example, for 12 months of exposure to PM_2.5_, the odds of developing anosmia at 6.0 µg/m^3^ was OR 0.79 (95% CI, 0.64-0.97); at 10.0 µg/m^3^, OR 1.42 (95% CI, 1.10-1.82); at 15.0 µg/m^3^, OR 2.03 (95% CI, 1.15-3.58).

**Table 2.** Conditional Logistic Regression Analyses for the Association Between Exposure to Air Pollution and Anosmia

Time exposed to PM_2.5_, mo	OR (95% CI)^a^		
	Model 1^b^	Model 2^c^	Model 3^d^
12	1.53 (1.16-2.02)	1.68 (1.25-2.26)	1.73 (1.28-2.33)
24	1.58 (1.21-2.07)	1.69 (1.27-2.24)	1.72 (1.30-2.29)
36	1.58 (1.23-2.03)	1.66 (1.27-2.16)	1.69 (1.30-2.19)
60	1.48 (1.15-1.90)	1.56 (1.20-2.03)	1.59 (1.22-2.08)

Abbreviations: OR, odds ratio; PM_2.5_, particulate matter with an aerodynamic diameter of no more than 2.5 μm.

^a^ ORs are based on 5-μg/m^3^ increase in PM_2.5_ exposure.

^b^ Adjusted for age, sex, race/ethnicity, and state.

^c^ Additionally adjusted for body mass index, current alcohol consumption status, current smoking status, and median household income.

^d^ Additionally adjusted for medical history of hypertension, diabetes, chronic obstructive pulmonary disease, and asthma.

**Figure.**
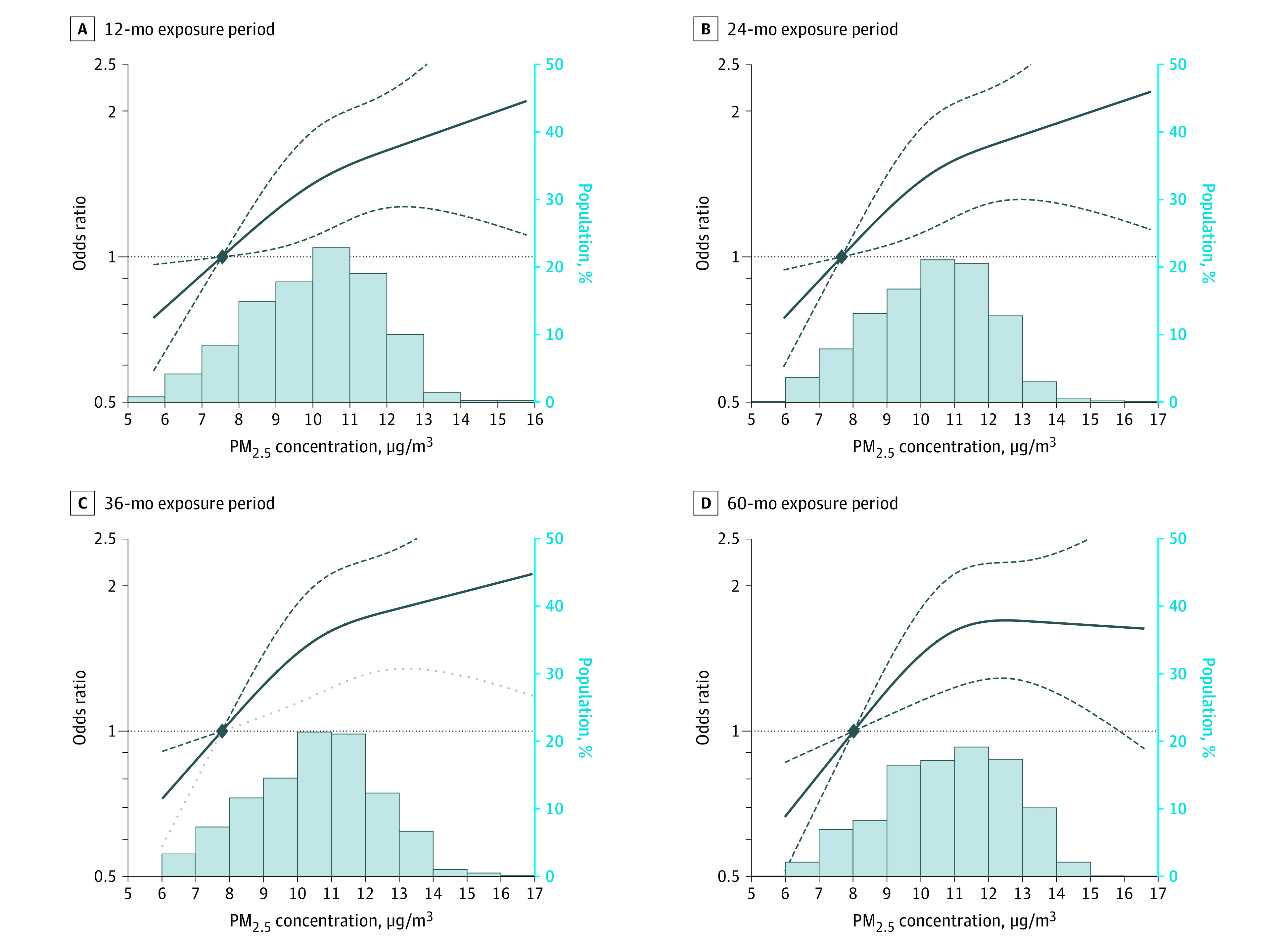
Odds Ratios (ORs) for Risks of Anosmia by the Level of Exposure to Particulate Matter With an Aerodynamic Diameter of No More Than 2.5 μm (PM_2.5_) Concentration in Each Exposure Period The dose-response curve was calculated using restricted cubic splines with knots at the 10th, 50th, and 90th percentiles of the distribution of 12-month PM_2.5_ concentrations. The reference exposure level was set at the 10th percentile of the distribution of 12-month PM_2.5_ concentrations (7.56 μg/m^3^). ORs were adjusted for age, sex, race/ethnicity, state, body mass index, current alcohol consumption status, current smoking status, median household income of zip code of individual's residence, and medical history of hypertension, diabetes, chronic obstructive pulmonary disease, and asthma.

## Discussion

To our knowledge, this study found the strongest association to date between long-term exposure to air pollution and anosmia. We observed a dose-dependent association between increasing concentrations of PM_2.5_ exposure and anosmia that persisted over 5 years of PM_2.5_ exposure, even after adjusting for confounding factors. The current findings suggest that even small increases in ambient PM_2.5_ exposure may be associated with anosmia, which has broad public health ramifications in the setting of increasing global urbanization. This study benefits from many strengths, including a robust patient data set and the use of a novel control matching strategy. We have also used unique deep learning neural network modeling to accurately estimate PM_2.5_ exposure to demonstrate realistic clinical implications of air pollution on olfactory function.

An often-overlooked human sensory function, olfaction is vital to the perception and experience of human life. Olfactory impairments are intrinsically associated with the experience of food, eating-related quality of life, and malnutrition.^1,28^ In fact, most subjective gustatory deficits are a manifestation of olfactory loss.^29^ Moreover, anosmia has been negatively associated with broad measures of quality of life, depression, anxiety, and cognitive impairment.^4,30,31,32,33,34,35^ Furthermore, in addition to inherent risks associated with the failure to detect toxins and environmental hazards, large population-based studies have demonstrated an association between olfactory disturbances and anosmia with measures of patient frailty and mortality.^5,36^ Because olfactory function declines with age and air pollution exposure is cumulative, our data are consistent with environmental determinants of chemosensory aging.^12,37^ Thus, air pollution may represent another ubiquitous risk factor for age-related sensory loss.

Determinants of olfactory function are multifactorial and can be broadly categorized into biology, individual experience, and environment.^38^ Although epigenetic and cultural differences in olfactory function have been described, the effect of environmental factors may be substantial.^39^ Several studies have attempted to capture the direct effect of pollution and industrialization on olfaction. Two studies^16,17^ from Mexico compared olfactory ability of residents from geographically similar locations that differed drastically in their level of air pollution. In each of these studies, residents from the less polluted environment outperformed residents from the more polluted city. Similar observations have been observed in other countries; for example, individuals from Dresden, Germany, were found to perform significantly worse than those from the Bolivian rainforest or the Cook Islands in the South Pacific, which are 2 significantly less polluted areas.^40,41^ Although confounding differences exist, these studies support a role for environmental determinants in affecting olfaction.

The pathophysiologic mechanism of olfactory loss associated with PM_2.5_ remains unclear. Evidence in the literature suggests that PM_2.5_ may create sinonasal inflammation, which may compromise the odorants’ ability to reach the olfactory cleft.^42^ Alternatively, pollution levels may result in mucosal inflammation, which affects the olfactory cleft.^43,44^ Indeed, nasal biopsies from residents of Mexico City demonstrated dysplastic epithelial changes compared with patients from the less polluted Isla Mujeres in Mexico, implying that cellular changes may occur without overt clinical manifestations.^45^ An alternative mechanism is that PM_2.5_ may cause direct insult to the olfactory neuroepithelium and olfactory bulb. There is also the possibility of direct nervous system insults, with increased levels of β-amyloid, cyclooxygenase-2, PM, and metals found in autopsies of patients with anosmia in both the olfactory bulb and frontal lobe, compared with control participants who experienced lower pollution levels.^20,46,47,48^ Additionally, the inhalation of ultrafine particles (PM <1 μm) may directly translocate along the olfactory nerve directly to the central nervous system.^49^ Overall, these results remain to be replicated and developed further in larger and more diverse human cohorts, different environments, and in animal models that can be manipulated.

The adverse effects of air pollution are pervasive and represent more serious implications for certain at-risk populations. The association between air pollution and more severe obstructive lower respiratory disease outcomes have been well-described,^50,51,52,53,54^ whereas more recent investigations have demonstrated the untoward effects of air pollution on the upper respiratory system.^43,44^ Although underlying respiratory disease may increase the relative risk of pollutant exposure, the associated health risks of air pollution are especially notable for lower-income, underserved, and minority communities, as they are often exposed to higher concentrations of potentially hazardous pollutants.^55,56,57^

Although substantially less is known regarding the association of pollutants with olfactory dysfunction compared with other diseases, there is increasing awareness regarding the importance of olfaction. Recently, COVID-19 has thrust olfaction into the spotlight as olfactory disturbances appear to be both a cardinal symptom and, in some cases, a debilitating consequence of the ongoing global pandemic. The inability to detect hazards, such as gas leaks or fires, represents the immediate implications of disruptions in olfactory function. In contrast, increased levels of depression, dietary changes, and impaired cognition may be associated with effects on patient frailty and mortality.^8,58,59^ Nonetheless, prior studies have demonstrated a persistent association of olfactory dysfunction and mortality even after correcting for dementia.^58^ In the context of increasing global urbanization and an aging population, the pervasive association of air pollution with olfaction are likely to increase.

In this study, we developed a novel satellite-based model to estimate long-term exposure to PM_2.5_ with high spatial and temporal resolution. This model enabled an estimation of individual-level exposure and overcame the issue of spatial coverage associated with the use of data collected solely from ground monitoring stations. We also used a convolutional layer in the neural network to estimate PM_2.5_ by aggregating variable values from nearby grid cells or monitoring sites. This approach is versatile and more accurate in modeling complex pollutant exposure.

The findings of this current investigation present many avenues for future research, including individual and population studies to better understand mechanisms of PM_2.5_-associated olfactory dysfunction. Also, air pollution is a mixture of pollutants, including PM_10_, nitrogen dioxide, black carbon, and ozone, which uniquely contribute to patients’ environment. However, air quality is often measured by individual components that may not reflect the actual effects of the mixture as a whole. It is also possible that individual components of the particulate matter, such as unique metals, may be associated with the prevalence of anosmia in this study population. Thus, further epidemiologic studies are required to examine the association of other components of air pollution, geographic regions, socioeconomic disparities, and personal activity on olfaction.

### Limitations

This study has limitations. Because of the study’s cross-sectional design, only prevalent anosmia case participants could be analyzed. Therefore, effect estimates are more likely to be associated with reverse causation and residual confounding. Additionally, although the air pollution exposure models had an excellent cross-validation performance, the model-estimated exposures are surrogates for personal exposure, which depend on daily activity patterns as well as workplace and commuting exposures. The models also failed to account for indoor air pollution and change in residential address during the study. Although personal monitoring would help to alleviate these potential sources of error, these strategies may introduce their own unique sources of bias and are not practical with a large study population. Additionally, it is also possible that not all causes of anosmia were fully accounted for in this study design because, in many instances, anosmia may be anecdotal, with a proportion of diagnoses occurring in the setting of viral or idiopathic insults. Furthermore, the robust association of pollutant exposure and anosmia demonstrated in this matched case-control investigation that persisted across all exposure levels and multivariate regressions suggests the potential bias from personal exposure was minimal.

## Conclusions

In this cross-sectional study, long-term exposure to increasing concentrations of PM_2.5_ exposure was associated with anosmia. This finding has broad implications for the association of a prevalent ambient air pollutant with a vital human sensory function.
